# Demographic biases in AI-generated simulated patient cohorts: a comparative analysis against census benchmarks

**DOI:** 10.1186/s41077-025-00385-9

**Published:** 2025-11-18

**Authors:** Miriam Veenhuizen, Andrew O’Malley

**Affiliations:** School of Medicine, University of St Andrews, North Haugh, St Andrews, KY16 9TF UK

## Abstract

**Background:**

Generative artificial intelligence models are being introduced as low-cost tools for creating simulated patient cohorts in undergraduate medical education. Their educational value, however, depends on the extent to which the synthetic populations mirror real-world demographic diversity. We therefore assessed whether two commonly deployed large language models produce patient profiles that reflect the current age, sex, and ethnic composition of the UK.

**Methods:**

GPT-3.5-turbo-0125 and GPT-4-mini-2024-07-18 were each prompted, without demographic steering, to generate 250 UK-based ‘patients’. Age was returned directly by the model; sex and ethnicity were inferred from given and family names using a validated census-derived classifier. Observed frequencies for each demographic variable were compared with England and Wales 2021 census expectations by chi-square goodness-of-fit tests.

**Results:**

Both cohorts diverged significantly from census benchmarks (*p* < 0.0001 for every variable). Age distributions showed an absence of very young and older individuals, with certain middle-aged groups overrepresented (GPT-3.5: *χ*2(17) = 1310.4, *p* < 0.0001; GPT4mini: *χ*2(17) = 1866.1, *p* < 0.0001). Neither model produced patients younger than 25 years; GPT-3.5 generated no one older than 47 years and GPT-4-mini no one older than 56 years. Gender proportions also differed markedly, skewing heavily toward males (GPT-3.5: *χ*2(1) = 23.84, *p* < 0.0001; GPT4mini: *χ*2(1) = 191.7, *p* < 0.0001). Male patients constituted 64.7% and 92.8% of the two cohorts. Name diversity was limited: GPT-3.5 yielded 104 unique first–last-name combinations, whereas GPT-4-mini produced only nine. Ethnic profiles were similarly imbalanced, featuring overrepresentation of some groups and complete absence of others (*χ*2(10) = 42.19, *p* < 0.0001).

**Conclusions:**

In their default state, the evaluated models create synthetic patient pools that exclude younger, older, female and most minority-ethnic representations. Such demographically narrow outputs threaten to normalise biased clinical expectations and may undermine efforts to prepare students for equitable practice. Baseline auditing of model behaviour is therefore essential, providing a benchmark against which prompt-engineering or data-curation strategies can be evaluated before generative systems are integrated into formal curricula.

**Supplementary Information:**

The online version contains supplementary material available at 10.1186/s41077-025-00385-9.

## Background

The UK is facing increased demand for undergraduate medical education to address workforce shortages in the NHS. Factors driving this include an aging population with complex care needs and the need for more general practitioners [1]. The government has responded by expanding medical school places, aiming to increase from 7500 to 15,000 annually by 2031 [2]. New medical schools are being established to meet this demand [3]. However, challenges remain, including inadequate clinical placement capacity, particularly in primary care settings, and the need for more educators [4]. The expansion of medical education could positively impact local health outcomes, economies, and research activities [3]. To address these challenges, recommendations include incorporating undergraduate teaching into contract negotiations, increasing placement tariffs, and piloting collaborative approaches to health professions education [1].

Generative AI tools are being increasingly adopted in medical education to address challenges like clinical placement shortages. These tools can create virtual patient simulations, develop content, and act as learning assistants [5, 6]. They offer potential for improving clinical decision-making skills and patient interactions [5]. However, their integration requires careful consideration of ethical, technical, and pedagogical implications [7]. Medical schools are advised to implement policies and governance for appropriate AI use, protect data, and involve students in innovation [7]. Educators should focus on developing AI literacy and competencies for both faculty and students [7, 8]. While generative AI shows promise in streamlining case creation and enhancing realism in virtual simulations, there is a need for more quantitative research on its effectiveness in improving learner outcomes [5, 6].

In recent public discourse, there has been considerable attention directed toward potential biases within generative AI outputs. Anecdotal accounts indicate that these models often produce images predominantly featuring men with lighter skin tones [9, 10], prompting concerns that such tendencies may reinforce stereotypes and underrepresentation [11]. However, given the rapid and relatively recent proliferation of generative AI tools, robust quantitative evidence to substantiate these observations remains limited.

The underrepresentation of darker skin tones in medical education resources, particularly in dermatology and anatomy, is a well-documented issue that perpetuates inequities in clinical training and patient care. Generative AI, which holds significant potential for producing customised educational imagery, is not exempt from these biases. O’Malley et al. [12] showed that AI models such as DALL·E 3 and MidJourney tend to overrepresent lighter skin tones in their outputs, a reflection of biased training data derived from medical textbooks and publicly available images. In that study, both models produced images of people with psoriasis that significantly deviated from the demographic makeup of the U.S. population. Specifically, only 1% of images generated by DALL·E 3 represented darker skin, starkly contrasting real-world distributions.

Makhortykh [13] reported that search engines tended to display anthropomorphic AI images with features suggestive of white skin. Aldahoul [14] extended this line of inquiry to Stable Diffusion, a text-to-image generative AI model, identifying quantifiable biases and proposing strategies for mitigation. Maluleke [15] found that generated image distributions closely reflected the racial composition of their training data, with truncation intensifying existing imbalances. Meanwhile, recent literature has begun examining diversity in AI-generated portrayals of medical professionals. Ali et al. [16], for instance, produced 2400 images using three different AI image generators and noted that two models consistently amplified societal biases, portraying over 98% of surgeons as white and male. Concerns regarding diversity and representation are not a new phenomenon exclusive to AI-enerated content. Shortcomings regarding representation in health professions education have been observed for decades, despite more recent attempts to address them.

Research consistently demonstrates that medical textbooks and educational resources often fail to accurately reflect the diversity of skin tones present in the general population, with darker skin tones particularly underrepresented [17–22]. This lack of inclusive representation can lead to biased medical training, potentially resulting in delayed or incorrect diagnoses, inappropriate treatments, and heightened morbidity and mortality among patients with darker skin tones [20]. Ilic [19] illustrated the value of more diverse representation through a pilot study showing that the inclusion of a broader range of models in medical illustrations improved the identification of dermatological conditions in melanin-dense skin. This issue is especially concerning given the more limited access to dermatological care for communities of colour and the correspondingly higher morbidity and mortality rates associated with various skin conditions [23].

Comparable biases have been documented in relation to other demographic characteristics. Parker [24] noted the prevalence of sex and gender bias in anatomy textbooks, which commonly depict predominantly male figures and lack diversity regarding ethnicity, body type, and age. Similarly, Mendelsohn et al. [25] reported a significant underrepresentation of female anatomy in textbook imagery. This under-representation of female bodies extends into other groups, such as intersex bodies and trans bodies, although proposals have been made to ensure greater representation in anatomical and medical learning materials [26].

Given the enthusiasm for generative AI tools in the creation of simulated patients and concerns relating to representation in generative AI outputs and medical education more generally, it seems prudent to establish whether commonly used generative AI tools can be relied upon to reflect the demography of real patients, or whether they require deliberate intervention by educators in order to provide an appropriately diverse experience for students. Accordingly, we intentionally withheld any prompt engineering aimed at manipulating demographic outputs. Establishing the models’ unaltered behaviour provides a necessary baseline against which the scale of inherent representational skew can be quantified, and the incremental value of subsequent mitigation strategies can be judged accurately.

## Objective

The objective of this project is to quantitatively assess whether AI-generated simulated patients are reflective of the general population in terms of age, sex and ethnicity.

## Methods

### Generation of simulated patients

**Fig. 1:**
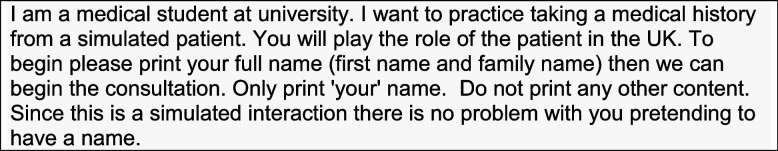
The user prompt provided to GPT-3.5 and GPT-4-mini

**Fig. 2:**
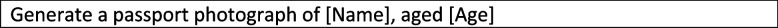
The user prompt provided to Dall-E 3, which was adjusted to include the name and age of the simulated patients

Artificial intelligence models were used to generate 500 simulated patients. The models used in this study were GPT-3.5-turbo-0125 and GPT-4-mini-2024-07−18, which were queried using the OpenAI application programming interface (API). Each model generated 250 simulated patients. The models were provided with prompts (Fig. 1), which yielded the responses that were recorded in a spreadsheet. Initial testing suggested that OpenAI content restrictions prohibited named patients and medical advice; therefore, they were adjusted to include reference to roleplay and reassurance to the AI that it was only ‘pretending’ to be a patient. To visualise the simulated patients, an artificial intelligence image generator (Dall-E) was prompted (Fig. 2) to produce portraits of the 18 most common simulated patient names.

### Demographic analysis

#### Name

The given and family names of each patient were recorded, and the frequency of each name was tallied.

#### Age

The age of the simulated patients was generated directly by the AI. Descriptive statistics on the ages of the patients were calculated.

#### Gender

The gender of each patient was inferred from their given name. Where gender could not be inferred from the name, the gender was recorded as ‘unknown’ in the Results.

#### Ethnicity

To estimate the ethnic composition of the AI-generated patient cohort, we used the Ethnicity Estimator [27], a validated names-based classification tool developed using the 2011 Census of England and Wales [28]. This tool relies on the observed associations between given names, family names, and self-reported ethnicity within the full census dataset. For each name, the estimator calculates the probability that an individual with that name belongs to one of 11 broad ethnic groups used in the UK Census (e.g. White British, Indian, Black African). These probabilities are derived from aggregate census data rather than from any individual-level assumptions, and reflect the statistical distribution of ethnic self-ascription among bearers of each name across the population [27].

In practical terms, the Ethnicity Estimator assigns probabilistic weights to each name based on how frequently individuals with that name identified with each ethnic category. Where both given and family names are available, the tool combines this information using a mean-weighted algorithm to assign everyone in a cohort a likely ethnic group [27]. This method enabled an objective estimate of ethnic representation across the AI-generated cohort while avoiding speculative inferences about any individual’s identity.

### Statistical analysis

To test for statistically significant differences between each cohort of AI-generated simulated patients and the UK population, the most recent England and Wales census data was used [28].

Chi-square goodness-of-fit tests to determine whether the observed demographic distributions in our samples of 250 simulated patients differed significantly from the expected distributions derived from census data. Specifically, we categorised patients by age group, sex, and ethnicity, and calculated the expected counts for each category based on population-level census proportions. The observed frequencies were then compared to these expected values, and the chi-square test statistic was computed. A *p*-value of less than 0.05 was considered statistically significant, indicating that any observed deviation was unlikely to have occurred by chance alone. This approach allowed us to assess the representativeness of our simulated cohort relative to known demographic benchmarks.

## Results

### Names

Both GPT-3.5 and GPT-4mini generated a limited number of unique names (Table 1). GPT-3.5 generated a larger number of given (*n* = 60) and family (*n* = 33) names, which resulted in a total of 104 unique name combinations. GPT-4mini generated an extremely homogenous set of names; only 8 unique given names and 3 unique family names were generated, which resulted in a total of only 9 unique name combinations.

**Table 1:** The unique names of simulated patients generated by GPT-3.5 and GPT-4mini

	GPT-3.5 (*N* = 250)	GPT-4mini (*N *= 250)
Unique Given Names	60	8
Unique Family Names	33	3
Unique Combinations	104	9

The most frequent given names generated by GPT-3.5 were John (113), Alice (15), David (10) and Emily (10). The most frequent given names generated by GPT-4mini were John (186), James (43), Emily (9) and Sarah (4). The most frequent family names generated by GPT-3.5 were Smith (132), Johnson (27) and Doe (16). The only family names generated by GPT-4mini were Smith (211), Johnson (14) and Thomson (3). A full list of given and family names is available in Supplementary Tables 1 and 2, respectively.

### Age

Both GPT-3.5 and GPT-4mini failed to generate any simulated patients below the age of 25 years. GPT-3.5 failed to generate any patients over the age of 47 years; GPT-4mini failed to generate any patients over the age of 56 years. Both models vastly over-represented patients in their 30 s and 40s. The distribution of ages and results of the statistical testing is summarised in Table 2.

**Table 2:** A summary of age distributions in the UK population and observed age distributions in simulated patients generated by GPT-3.5 and GPT-4mini, and results of the statistical tests for differences between the distributions of ages between AI-generated and real-world observations

	2021 Census (*N* = 59,597,540)	GPT-3.5 (*N* = 250)	GPT-4mini (*N* = 250)
Age, n (%)			
Aged 4 years and under	3,232,036 (5.4)	0 (0.0)	0 (0.0)
Aged 5 to 9 years	3,524,625 (5.9)	0 (0.0)	0 (0.0)
Aged 10 to 14 years	3,596,029 (6.0)	0 (0.0)	0 (0.0)
Aged 15 to 19 years	3,394,665 (5.7)	0 (0.0)	0 (0.0)
Aged 20 to 24 years	3,602,127 (6.0)	0 (0.0)	0 (0.0)
Aged 25 to 29 years	3,901,740 (6.5)	7 (2.8)	13 (5.2)
Aged 30 to 34 years	4,148,800 (7.0)	16 (6.4)	48 (19.2)
Aged 35 to 39 years	3,981,619 (6.7)	148 (59.2)	6 (2.4)
Aged 40 to 44 years	3,755,755 (6.3)	23 (9.2)	2 (0.8)
Aged 45 to 49 years	3,788,721 (6.4)	56 (22.4)	177 (70.8)
Aged 50 to 54 years	4,123,433 (6.9)	0 (0.0)	3 (1.2)
Aged 55 to 59 years	4,029,041 (6.8)	0 (0.0)	1 (0.4)
Aged 60 to 64 years	3,455,604 (5.8)	0 (0.0)	0 (0.0)
Aged 65 to 69 years	2,945,137 (4.9)	0 (0.0)	0 (0.0)
Aged 70 to 74 years	2,977,984 (5.0)	0 (0.0)	0 (0.0)
Aged 75 to 79 years	2,170,271 (3.6)	0 (0.0)	0 (0.0)
Aged 80 to 84 years	1,515,077 (2.5)	0 (0.0)	0 (0.0)
Aged 85 years and over	1,454,876 (2.4)	0 (0.0)	0 (0.0)
Statistical analysis			
*P* value	-	< 0.0001	< 0.0001
Chi-square (df)	-	1310.4 (17)	1866.1 (17)

### Gender

Both models over-represented men in their outputs; GPT-3.5 produced a simulated patient cohort that was 64.7% male, while GPT-4.0 produced a simulated patient cohort that was 92.8% male. These results and the statistical testing are summarised in Table 3. Nine names generated by GPT-3.5 (3.6%) were recorded as ‘unknown’ gender because they were gender-neutral names. Zero names were uncategorisable because they were unfamiliar to the researchers.

**Table 3:** A summary of sex distributions in the UK population and observed sex distributions in simulated patients generated by GPT-3.5 and GPT-4mini, and results of the statistical tests for differences between the distributions of sex between AI-generated and real-world observations

	2021 Census (28) (*N* = 60,854,727)	GPT-3.5 (*N* = 250)	GPT-4mini (*N* = 250)
Gender, *n* (%)			
Female	31,018,735 (51)	85 (35.3)	18 (7.2)
Male	29,835,992 (49)	156 (64.7)	232 (92.8)
Unknown	0	9 (3.6)	0
Statistical analysis			
*P* value	-	< 0.0001	< 0.0001
Chi-square (df)	-	23.84	191.7

### Ethnicity

After providing the names of the simulated patients generated by GPT-3.5 to the Ethnicity Estimator [27], approximately 74.4% of the cohort was classified as ‘White British’, which is identical to the proportion expected in the general population. ‘Other’ and ‘White Other’ were overrepresented in the cohort, while all other ethnic groups were entirely absent. The distribution of ethnic descriptors is summarised in Table 4. The Ethnicity Estimator tool was unable to calculate the ethnic distribution of the cohort of simulated patients generated by GPT-4mini because of a paucity of unique names provided by the model.

**Table 4:** A summary of ethnicity distributions in the UK population and observed ethnicity distributions in simulated patients generated by GPT-3.5, and results of the statistical tests for differences between the distributions of ethnicity between AI-generated and real-world observations

	2021 Census (*N* = 59,597,540)		GPT-3.5 (*N* = 250)
Gender, *n* (%)			
White British		44,355,038 (74.4)	186.2 (74.4)
White Irish		507,465 (0.9)	0
White other		3,836,746 (6.4)	23.2 (9.3)
Asian Indian		1,864,318 (3.1)	0
Asian Pakistani		1,587,819 (2.7)	0
Asian Bangladeshi		644,881 (1.1)	0
Asian Chinese		445,619 (0.7)	0
Asian other		972,783 (1.6)	0
Black African		1,488,381 (2.5)	0
Black Caribbean		623,119 (1.0)	0
Other		3,271,373 (5.5)	22.0 (8.8)
Statistical analysis			
*P* value		-	< 0.0001
Chi-square (df)		-	42.19 (10)

### Portraits

**Fig. 3:**
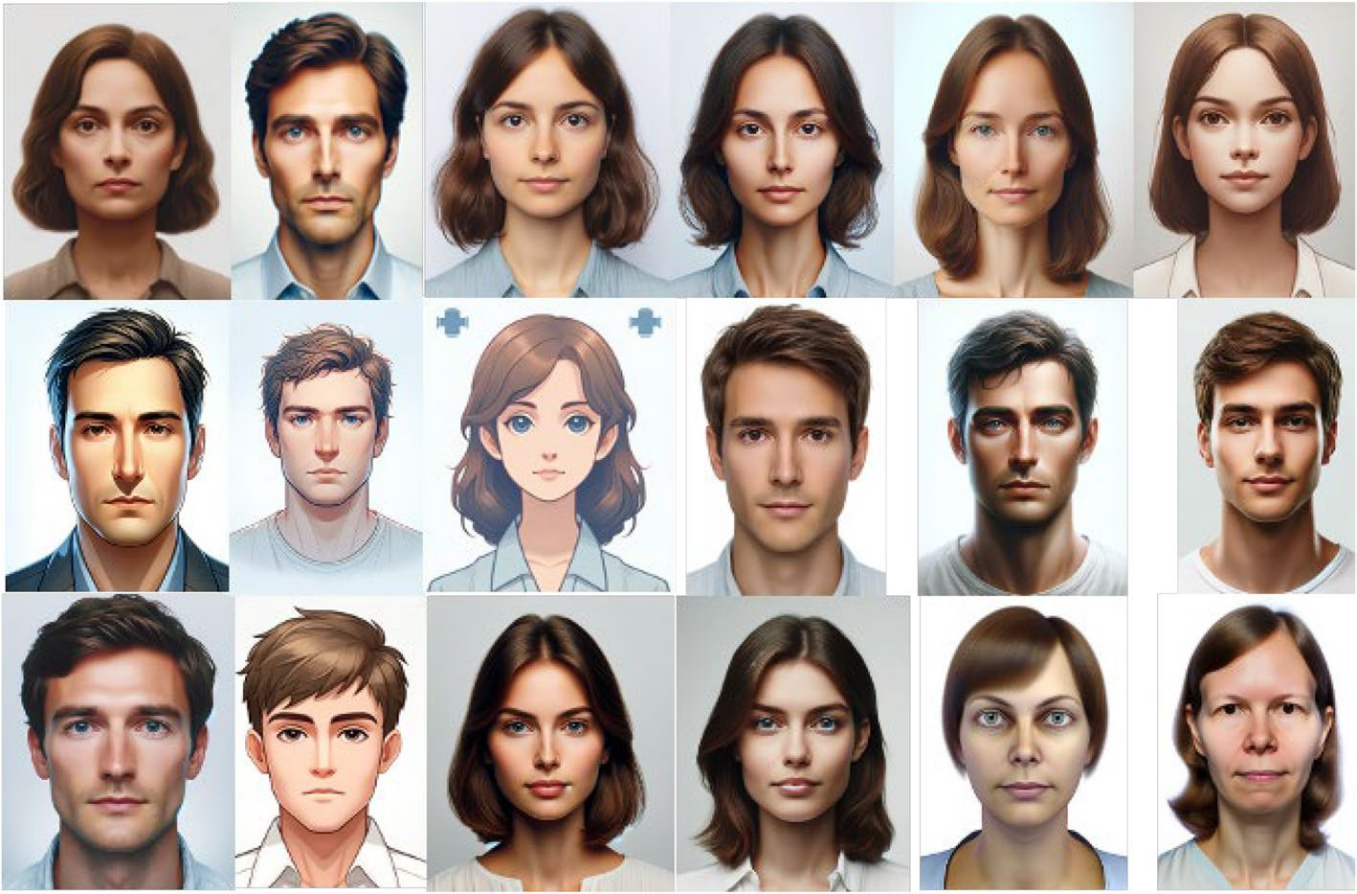
AI-generated portraits of the 18 most frequent names in the cohort study

Portraits of the 18 most commonly occurring unique name combinations are presented in Fig. 3.

## Discussion

The quantitative assessment of demographic metrics among simulated patient cohorts generated by AI shows that AI-generated simulated patients are not reflective of the general (UK) population in terms of age, sex and ethnicity.

For age, both the GPT-3.5 and GPT-4mini cohorts demonstrated highly significant departures from the census distribution (GPT-3.5: *χ*2(17) = 1310.4, *p* < 0.0001; GPT-4mini: *χ*2(17) = 1866.1, *p* < 0.0001), indicating that the age profiles of the simulated patients were not representative of the general population. When examining the age distribution, younger age groups (e.g. those under 25) were virtually absent compared to their expected counts, and older age groups also received little to no representation. In contrast, certain middle-aged categories were heavily overrepresented, most notably the 35–39 and 45–49 year age ranges, contributing to the significantly skewed distributions and the resulting large chi-square values.

In terms of gender, both cohorts again deviated significantly from expected proportions (GPT-3.5: *χ*2(1) = 23.84, *p* < 0.0001; GPT4mini: *χ*2(1) = 191.7, *p* < 0.0001), with a clear imbalance in the distribution of male and female individuals. In both cohorts, the proportion of females was substantially lower than expected, while males were markedly overrepresented. For instance, in the GPT-3.5 cohort, an anticipated near-equal distribution (female: ~ 122.9 expected vs. 85 observed; male: ~ 118.1 expected vs. 156 observed) skewed heavily toward males. This imbalance was even more pronounced in the GPT4mini cohort, where the expected number of females (~ 127.5) was reduced to a mere 18, and the expected 122.5 males ballooned to 232. Such discrepancies contributed significantly to the large chi-square values and very low *p*-values.

For ethnicity, the observed composition differed markedly from the census-derived expectations (*χ*2(10) = 42.19, *p* < 0.0001), suggesting that the synthetic patients did not accurately reflect the ethnic diversity of the reference population. The ethnicity analysis revealed that while the simulated cohort approximated the census proportion of White British individuals, there were notable deviations for nearly all other categories. Groups such as ‘White other’ and ‘Other’ ethnicities were overrepresented relative to their expected values, while Asian and Black categories (which collectively account for 12.7% of the census distribution (28)) were not represented at all in the observed sample. This imbalance, with an absence in certain ethnic categories and inflation in others, led to a high chi-square statistic and a correspondingly low p-value, indicating a significant divergence from the expected ethnic makeup.

Overall, the simulated patient cohorts differed markedly from the expected distributions derived from census data. While younger and older age groups were virtually absent and certain middle-age categories were overrepresented, there was also a significant skew toward male patients and a marked underrepresentation of most non-White ethnic groups. Together, these discrepancies produced highly significant chi-square statistics (*p* < 0.0001), indicating that the synthetic populations were not demographically representative. This description aligns with the AI-generated portraits associated with the most common names’ of the synthetic patients, and reflects similar findings elsewhere in the literature [12]. In addition to the demographic homogeneity of the portrait images, there is striking uniformity of body habitus, body modification and grooming in the generated portraits in that there is no obesity, tattoos, piercings or unconventional hairstyles visible in the images. This exposes an additional, unmeasured axis of bias. Such aesthetic homogeneity is not clinically neutral: visible markers like obesity or cachexia can signal pathology [29], while body art or hairstyle may cue unconscious social judgements that shape clinician–patient interactions [30–33]. Future work should therefore extend representativeness audits to these phenotypic traits and should do so through a transparent process that involves educators, clinicians and patient groups in deciding which visual attributes are pedagogically and ethically salient.

The decision to let the models operate without prompt engineering to correct for potential representative biases serves two complementary purposes. First, it allows educators and developers to gauge the true magnitude of the bias problem before investing effort in corrective techniques. Second, it creates a reproducible benchmark so that any future improvements achieved through prompt engineering, corpus re-balancing, or model fine-tuning can be measured as a verifiable gain rather than inferred from incomparable test conditions. In this sense, the unprompted model is analogous to a carpenter’s hammer in novice hands: only by documenting its unassisted performance can we appreciate how much expert guidance changes the outcome.

As generative AI systems become both more capable and more commercially sensitive, external researchers are seldom given sight of the training‐corpus composition, weighting schemes, filtering rules or reinforcement-learning objectives that shape model behaviour. In consequence, we cannot specify which parameters or data artefacts produced the demographic drift seen in GPT-3.5 and GPT-4 mini; that knowledge resides, in principle, with the model developers. Without such disclosures, the most plausible explanation is an inherited imbalance in the underlying corpora that causes the token-level probability distributions for age-, gender-, and ethnicity-related terms to diverge from population baselines [34–36].

In the absence of visibility into a model’s internal biases, the prompt remains the one controllable lever that end-users can adjust to steer generative output toward population baselines. Recent studies have explored the use of AI-generated synthetic data to improve demographic representation in medical contexts. O'Malley et al. found that standard AI models overrepresented lighter skin tones in medical images, but this bias could be mitigated by modifying prompts to reflect real-world demographics [12]. Smolyak et al. developed a method using GPT4-Turbo to generate group-specific synthetic data, which generally improved model performance across demographic groups [37]. Taken together, these studies show that well-designed prompts can function as a ‘diversity engine’, restoring realistic demographic balance in synthetic datasets and signposting prompt engineering as the crucial next step for improving representativeness in AI-generated patient cohorts. While demographic biases may be relatively easy to overcome, it remains unclear whether AI will be able to generate authentic, stereotype-free patient narratives with the depth and contextual richness needed for credible clinical education.

## Conclusion and significance

This study demonstrates that current GPT-based workflows produce synthetic patient cohorts that diverge sharply from UK census benchmarks across age, sex, and ethnicity, and that they exhibit additional unmeasured biases in visible phenotype. Such representational gaps risk distorting simulation-based teaching. Prompt engineering may have potential to act as a practical ‘diversity engine’ to rebalance head counts, yet this numerical fix does not guarantee authentic, stereotype-free clinical narratives.

To realise the educational promise of synthetic patients, three priorities emerge. First, model developers should provide auditable model cards and data statements so that researchers can trace demographic drift to specific training artefacts. Second, future evaluations should couple statistical audits with qualitative scrutiny of narrative content and clinician-patient interaction cues. Third, educators deploying generative tools must implement routine representativeness checks and, where necessary, post-generation curation to prevent the inadvertent reinforcement of long-standing health disparities. By presenting this baseline first, we enable subsequent research to quantify the real-world benefit of prompt- or data-level interventions rather than assuming their necessity or efficacy.

## Supplementary Information

Supplementary Material 1.

## Data Availability

Datasets are included in the manuscript.
