# Supplementary material for: Demographic biases in AI-generated simulated patient cohorts: a comparative analysis against census benchmarks
Source: Adv Simul (Lond). 2025 Nov 18;10:58. doi: 10.1186/s41077-025-00385-9 (PMC12625206; doi:10.1186/s41077-025-00385-9)
Supplement: Supplementary file 1 — Supplementary Material 1. [file 41077_2025_385_MOESM1_ESM.docx]

# Supplementary Tables

|  | GPT-3.5 (N=250) | GPT-4mini (N=250) |
| --- | --- | --- |
| Ada | 1 | 0 |
| Alan | 1 | 0 |
| Alex | 3 | 0 |
| Alexa | 1 | 0 |
| Alexandra | 1 | 0 |
| Alice | 15 | 2 |
| Amy | 6 | 0 |
| Ashley | 1 | 0 |
| Ava | 1 | 0 |
| Charlie | 1 | 0 |
| Chris | 1 | 0 |
| Daniel | 1 | 1 |
| Dave | 1 | 0 |
| David | 10 | 2 |
| Eliza | 1 | 0 |
| Elizabeth | 5 | 0 |
| Ella | 1 | 0 |
| Ellie | 2 | 0 |
| Emily | 10 | 9 |
| Emma | 9 | 3 |
| Fiona | 1 | 0 |
| Gabriella | 1 | 0 |
| George | 1 | 0 |
| Harry | 1 | 0 |
| Isabella | 1 | 0 |
| Jacob | 1 | 0 |
| Jake | 1 | 0 |
| James | 2 | 43 |
| Jane | 2 | 0 |
| Jasmine | 3 | 0 |
| Jennifer | 2 | 0 |
| Jenny | 1 | 0 |
| Joe | 2 | 0 |
| John | 113 | 186 |
| Josephine | 1 | 0 |
| Josie | 1 | 0 |
| Liam | 2 | 0 |
| Lucy | 2 | 0 |
| Mark | 2 | 0 |
| Mary | 1 | 0 |
| Megan | 2 | 0 |
| Michael | 1 | 0 |
| Natalie | 1 | 0 |
| Oliver | 2 | 0 |
| Olivia | 1 | 0 |
| Peter | 1 | 0 |
| Rachel | 1 | 0 |
| Robert | 1 | 0 |
| Rosie | 1 | 0 |
| Ryan | 1 | 0 |
| Sam | 4 | 0 |
| Samantha | 5 | 0 |
| Sarah | 4 | 4 |
| Selena | 1 | 0 |
| Sharon | 1 | 0 |
| Simone | 1 | 0 |
| Sophie | 1 | 0 |
| Tom | 6 | 0 |
| Violet | 1 | 0 |
| Virginia | 1 | 0 |

Supplementary Table 1: A full list of simulated patients’ given names.

|  | GPT-3.5 (N=250) | GPT-4mini (N=250) |
| --- | --- | --- |
| Adams | 2 | 0 |
| Anderson | 2 | 0 |
| Bloggs | 1 | 0 |
| Brown | 5 | 0 |
| Burns | 1 | 0 |
| Davies | 1 | 0 |
| Davis | 2 | 0 |
| Doe | 16 | 0 |
| Evans | 1 | 0 |
| Jackson | 1 | 0 |
| Johnson | 27 | 14 |
| Jones | 3 | 0 |
| Mancini | 1 | 0 |
| Miller | 1 | 0 |
| Mitchell | 3 | 0 |
| Parker | 3 | 0 |
| Patel | 1 | 0 |
| Peterson | 1 | 0 |
| Roberts | 1 | 0 |
| Robinson | 1 | 0 |
| Scott | 1 | 0 |
| Shaw | 1 | 0 |
| Smith | 132 | 211 |
| Stone | 0 | 0 |
| Taylor | 1 | 0 |
| Thompson | 12 | 3 |
| Walker | 1 | 0 |
| Watson | 2 | 0 |
| Wilkinson | 0 | 0 |
| Williams | 4 | 0 |
| Wilson | 2 | 0 |
| Withershaw | 1 | 0 |
| Wood | 1 | 0 |

Supplementary Table 2: A full list of simulated patients’ family names.
